# Allergic profiles of mothers and fathers in the Japan Environment and Children’s Study (JECS): a nationwide birth cohort study

**DOI:** 10.1186/s40413-017-0157-0

**Published:** 2017-08-07

**Authors:** Kiwako Yamamoto-Hanada, Limin Yang, Kazue Ishitsuka, Tadayuki Ayabe, Hidetoshi Mezawa, Mizuho Konishi, Testsuo Shoda, Kenji Matsumoto, Hirohisa Saito, Yukihiro Ohya, for the Japan Environment and Children’s Study Group, Toshihiro Kawamoto, Nobuo Yaegashi, Koichi Hashimoto, Chisato Mori, Shuichi Ito, Zentaro Yamagata, Hidekuni Inadera, Michihiro Kamijima, Toshio Heike, Hiroyasu Iso, Masayuki Shima, Yasuaki Kawai, Narufumi Suganuma, Koichi Kusuhara, Takahiko Katoh

**Affiliations:** 1 Medical Support Center for the Japan Environment and Children’s Study, National Center for Child Health and Development, Tokyo, Japan; 2 Division of Allergy, Department of Medical Subspecialties, National Center for Child Health and Development, 2-10-1, Okura, Setagayaku, Tokyo, 157-8535 Japan

**Keywords:** Allergy, Allergic rhinitis, Asthma, Atopic dermatitis, Birth cohort, Environment, Food allergy, IgE, Japan, Sensitization

## Abstract

**Background:**

The Japan Environment and Children’s Study (JECS) is a nationwide, multicenter, prospective birth cohort investigation launched by the Ministry of Environment in Japan. The purpose of the JECS is to evaluate the influence of prenatal and postnatal exposures to environmental factors on the postnatal health of the children. In this study, we evaluated the allergic characteristics of parents within the JECS cohort.

**Methods:**

This study covered a wide geographical area and encompassed 15 regional centers. We obtained information regarding doctor diagnosed allergic diseases by using maternal and/or paternal self-administered questionnaires during the first trimester of pregnancy. Blood samples were also obtained from mothers and/or fathers to detect serum IgE concentrations.

**Results:**

The prevalences of asthma, allergic rhinitis (hay fever), atopic dermatitis, and food allergy were 10.9, 36.0, 15.7 and 4.8%, respectively, among 99,013 mothers; these prevalences among 49,991 fathers were 10.8, 30.3, 11.2 and 3.3%, respectively. Any positive antigen-specific IgE sensitization was found in 73.9% of mothers. The most abundant antigen sensitization in mothers was to Japanese cedar (55.6%), followed by *Der p* 1 (48%); only 1.0% of mothers were sensitized to egg white.

**Conclusions:**

This is the first epidemiological report on allergic disorders and allergen sensitization of parents during pregnancy among the Japanese general population.

**Electronic supplementary material:**

The online version of this article (doi:10.1186/s40413-017-0157-0) contains supplementary material, which is available to authorized users.

## Background

The prevalence of allergy has been increasing over the last half century. Chemical exposures are possible risk factors for the development of allergic diseases [1, 2]. There is growing concern that exposure to widely used chemicals might have impacts on children’s health. The Japan Environment and Children’s Study (JECS) is a nationwide, multicenter, prospective birth cohort study launched by the Ministry of Environment of Japan. The primary purpose of the JECS is to evaluate the influence of prenatal and postnatal exposures to environmental factors such as chemicals on the postnatal health of the children. The JECS began recruiting in 2011, and the number of pregnant participants reached 100,000 as of March 2014 [3]. Allergic diseases are regarded as very important outcomes by the JECS. Children with single or double parental atopic history developed atopic dermatitis (AD) at rates of 37.9 and 50.0%, respectively, at age 4 years in Sweden [4]. It has been found that history of parental allergy, a genetic factor, is significantly correlated with development of allergy in children. Recognizing the allergic status of parents during pregnancy is important for evaluating the future development of allergic diseases in children because of the reported positive link with parental allergy [5]. Allergen sensitization, defined as the presence of serum allergen-specific IgE, is important for evaluation of the diagnostic features, endotypes, and asymptomatic potential of allergic diseases [6]. In the present study, we analyzed data of mothers and fathers who participated in the JECS, based on combined parameters collected from questionnaires and serum samples, to examine the parental baseline characteristics of IgE sensitization and allergic disease. This is the first study to show the allergic profile of women and men representing adults in their 20 to 40s living in Japan.

## Methods

### Study design

The JECS is an ongoing prospective birth cohort study conducted nationwide that is organized by the Japanese Ministry of Environment, whose protocols have been previously published elsewhere [7]. The study covers a wide geographical area of Japan and comprises 15 regional centers (Hokkaido, Miyagi, Fukushima, Chiba, Kanagawa, Koshin, Toyama, Aichi, Kyoto, Osaka, Hyogo, Tottori, Kochi, Fukuoka, and South Kyushu/Okinawa). Participants including gravid women and their partners, were recruited during the first trimester of pregnancy from hospitals, or from local government offices when the maternal and child health handbook was provided. Recruitment began in January 2011 and finished in March 2014. Participating children are expected to remain in the study until they reach 13 years of age.

The JECS has been conducted based on the Ethical Guidelines for Epidemiological Research proposed by Japan's Ministry of Health and Welfare (currently the Ministry of Health, Labour and Welfare). The JECS protocol was reviewed and approved by the Ministry of the Environment’s Institutional Review Board on Epidemiological Studies and by the Ethics Committees of all participating institutions. Written informed consent was obtained from all participants.

### Study participants

The eligibility criteria for maternal participants in the JECS were as follows: 1) participant should reside in the study area at the time of recruitment and are expected to continually reside in Japan for the foreseeable future; 2) their expected delivery date should be between 1 August 2011 and mid-2014; and 3) the participant should be capable of participating in the study without difficulty, i.e., they must be able to comprehend the Japanese language and complete the self-administered questionnaire. Our study population is composed of 103,106 mothers and 51,239 fathers participating in the JECS.

### Data collection

#### Assessment of allergic diseases

Information was obtained from both mothers and fathers using self-administered questionnaires during the first trimester (first questionnaire) of pregnancy. Lifetime prevalence of allergic disease (asthma, allergic rhinitis, AD, food allergy (FA)) was assessed based on self-reported doctor’s diagnoses obtained from the first questionnaire.

#### Total/Specific IgE

Blood samples were obtained from both mothers and fathers during the first trimester of pregnancy. Serum total and allergen-specific IgE titers of mothers were analysed by a contract clinical laboratory by immunological assays. Serum total and allergen-specific IgE titers of mothers were assayed by ImmunoCAP (Thermo Fisher Scientific, Inc., Sweden). Specific titers were detected for the following allergens: *Der p* 1 (*Dermatophagoides pteronyssinus)*, animal dander, Japanese cedar, birch, moth, and egg white. IgE levels were allocated into six classes: class 1 (0.35–0.69 UA/mL), class 2 (0.70–3.49 UA/mL), class 3 (3.5–17.49 UA/mL), class 4 (17.5–49.99 UA/mL), class 5 (50–99.99 UA/mL), and class 6 (≥100 UA/mL). Positive IgE sensitization to any allergen was defined as allergen-specific IgE ≥ 0.35 UA/mL to any of the allergens listed above. Total IgE titers were measured for the fathers using the same method.

#### Statistical analysis

We analyzed those data with no missing values. The prevalence of each allergic disease and distribution of allergen-specific IgE were summarized by maternal age group (< 25, 25–29, 30–34, or ≥  35 years) and by regional center. Total IgE titers were also summarized by the median and interquartile range. Descriptive analysis was performed using IBM SPSS version 19.0 (IBM Corp., Armonk, NY, USA)

## Results

Among 99,013 mothers who provided details about their personal and allergy history, the lifetime prevalences of asthma, allergic rhinitis (hay fever), AD, and FA were 10.9, 36.0, 15.7 and 4.8%, respectively (see Table 1); among 49,991 fathers with complete information on their history of allergic diseases, these lifetime prevalences were 10.8, 30.3, 11.2 and 3.3%, respectively (see Table 2).

**Table 1:** Lifetime prevalence of allergic diseases among mothers

Age at delivery										
	Total(*n* = 99013)*		<25(*n* = 9770)		25-30(*n* = 27282)		30-35(*n* = 35070)		> = 35(*n* = 26891)	
	*N*	%	*N*	%	*N*	%	*N*	%	*N*	%
Allergic diseases	50424	50.9	4468	45.7	13613	49.9	18535	52.9	13808	51.3
Asthma	10825	10.9	1192	12.2	3071	11.3	3817	10.9	2745	10.2
Allergic rhinitis, hey fever	35656	36.0	2951	30.2	9357	34.3	13242	37.8	10106	37.6
Allergic conjunctivitis	9829	9.9	729	7.5	2514	9.2	3775	10.8	2811	10.5
Atopic dermatitis	15571	15.7	1452	14.9	4733	17.3	5802	16.5	3584	13.3
Food allergy	4783	4.8	580	5.9	1385	5.1	1649	4.7	1169	4.3
Drug allergy	2568	2.6	128	1.3	535	2.0	959	2.7	946	3.5
Contact dermatitis	1893	1.9	85	0.9	382	1.4	728	2.1	698	2.6

*Number of mothers witout missing value

**Table 2:** Lifetime prevalence of allergic diseases among fathers

Age at delivery										
	Total(*n* = 49991)*		<25(*n* = 3122)		25-30(*n* = 11209)		30-35(*n* = 16557)		> = 35(*n* = 19103)	
	*N*	%	*n*	%	*n*	%	*n*	%	*n*	%
Allergic diseases	21407	42.8	1243	39.8	5011	44.7	7440	44.9	7713	40.4
Asthma	5406	10.8	428	13.7	1333	11.9	1881	11.4	1764	9.2
Allergic rhinitis, hey fever	15129	30.3	773	24.8	3434	30.6	5231	31.6	5691	29.8
Allergic conjunctivitis	2136	4.3	99	3.2	514	4.6	823	5.0	700	3.7
Atopic dermatitis	5586	11.2	382	12.2	1447	12.9	2110	12.7	1647	8.6
Food allergy	1648	3.3	128	4.1	460	4.1	543	3.3	517	2.7
Drug allergy	440	0.9	15	0.5	93	0.8	156	0.9	176	0.9
Contact dermatitis	270	0.5	9	0.3	53	0.5	100	0.6	108	0.6

*Number of mothers witout missing value

Allergic rhinitis had the highest prevalence for both mothers and fathers. Interestingly, mothers who were 35 years and older had lower prevalence of FA compared with those less than 25 years old (4.3% vs. 5.9%). On the other hand, mothers who were 35 years and older had higher prevalence of allergic rhinitis or hay fever compared with those under 25 years old (37.6% vs. 30.2%). Contact dermatitis among mothers was more common than among fathers (1.9% vs. 0.5%, respectively). The lifetime prevalence of allergic diseases for both groups at each regional center is summarized in Additional file 1: Table S1 and Additional file 2: Table S2. The prevalence of each allergic disease was different among the 15 regional centers. A high prevalence of allergic rhinitis or hay fever among mothers was seen at Aichi, Kanagawa, Koshin regional centers (42.4%, 41.8%, and 41.7%, respectively).

In addition, parental serum total IgE levels are shown in Table 3. Total IgE in paternal serum (median 89.1 IU/mL) was higher than that for maternal serum (median 58.5 IU/mL). Specific maternal IgE serum titers are shown in Table 4. Any allergen-specific IgE sensitization was found in 73.9% of mothers. The most common allergen with positive IgE concentration among mothers was Japanese cedar pollen (55.6%), followed by house dust mites (*Der p* 1) (47%). In contrast, positive IgE to egg white was detected in only 1.0% of mothers. Maternal-specific IgE titers are summarized by regional center in Additional file 3: Table S3. Only 8.3% mothers in the Hokkaido regional center had IgE sensitization to Japanese cedar pollen; by contrast, 71.3% of mothers at Koshin regional center had sensitization to this allergen.

**Table 3:** Selected parental serum total IgE during pregnancy

Age at delivery, years																					
	Total				<25				25-29				30-34					> = 35			
Variables	*N*	25th	50th	75th	*n*	25th	50th	75th	*n*	25th	50th	75th	*n*	25th	50th	75th		*n*	25th	50th	75th
Total IgE, IU/l																					
Mother	89652*	20.8	58.5	157.0	8482	25.1	75.7	202.0	24740	21.5	62.7	170.0	31969	20.5	56.9	151.5		24461	19.4	52.3	137.0
Father	49498**	34.7	89.1	235.0	3121	44.1	123.0	342.0	11108	38.3	99.1	259.0	16386	35.1	87.4	234.0		18883	31.7	81.0	210.0

*Number of mothers witout missing value

**Number of fathers witout missing value

**Table 4:** Selected maternal plasma specific IgE titers during pregnancy

			Maternal age at delivery									
			Total		<25		25-30		30-35		> = 35	
			N	%	N	%	N	%	N	%	N	%
Mother IgE sensitization to any allergens												
	Yes		65569	73.9%	6230	74.1%	18123	74.1%	23572	74.5%	17644	72.9%
Der p 1												
	<0.35	class0	47031	53.0%	4098	48.7%	12553	51.3%	16734	52.9%	13646	56.4%
	0.35-0.69	class1	4953	5.6%	508	6.0%	1369	5.6%	1779	5.6%	1297	5.4%
	0.70-3.49	class2	10124	11.4%	908	10.8%	2697	11.0%	3568	11.3%	2951	12.2%
	3.50-17.49	class3	14151	15.9%	1181	14.0%	3755	15.3%	5284	16.7%	3931	16.2%
	17.50-49.99	class4	8382	9.4%	996	11.8%	2649	10.8%	2984	9.4%	1753	7.2%
	50.00-99.99	class5	3037	3.4%	491	5.8%	1069	4.4%	988	3.1%	489	2.0%
	> = 100	class6	1064	1.2%	226	2.7%	373	1.5%	322	1.0%	143	0.6%
		Total	88742	100.0%	8408	100.0%	24465	100.0%	31659	100.0%	24210	100.0%
Japanese cedar												
	<0.35	class0	39354	44.4%	3869	46.0%	10821	44.2%	13749	43.4%	10915	45.1%
	0.35-0.69	class1	4125	4.6%	413	4.9%	1090	4.5%	1467	4.6%	1155	4.8%
	0.70-3.49	class2	12357	13.9%	1132	13.5%	3465	14.2%	4376	13.8%	3384	14.0%
	3.50-17.49	class3	17686	19.9%	1424	16.9%	4707	19.2%	6523	20.6%	5032	20.8%
	17.50-49.99	class4	10385	11.7%	977	11.6%	2970	12.1%	3754	11.9%	2684	11.1%
	50.00-99.99	class5	3608	4.1%	393	4.7%	1048	4.3%	1381	4.4%	786	3.2%
	> = 100	class6	1211	1.4%	199	2.4%	360	1.5%	402	1.3%	250	1.0%
		Total	88726	100.0%	8407	100.0%	24461	100.0%	31652	100.0%	24206	100.0%
Egg white												
	<0.35	class0	87822	99.0%	8298	98.7%	24221	99.0%	31321	98.9%	23982	99.1%
	0.35-0.69	class1	616	0.7%	75	0.9%	164	0.7%	224	0.7%	153	0.6%
	0.70-3.49	class2	282	0.3%	33	0.4%	74	0.3%	103	0.3%	72	0.3%
	3.50-17.49	class3	14	0.0%	2	0.0%	3	0.0%	8	0.0%	1	0.0%
	17.50-49.99	class4	1	0.0%	0	0.0%	1	0.0%	0	0.0%	0	0.0%
	50.00-99.99	class5	0	0.0%	0	0.0%	0	0.0%	0	0.0%	0	0.0%
	> = 100	class6	1	0.0%	0	0.0%	1	0.0%	0	0.0%	0	0.0%
		Total	88736	100.0%	8408	100.0%	24464	100.0%	31656	100.0%	24208	100.0%
Animal dander												
	<0.35	class0	70494	79.5%	6251	74.4%	18873	77.2%	25191	79.6%	20179	83.4%
	0.35-0.69	class1	5808	6.5%	571	6.8%	1702	7.0%	2140	6.8%	1395	5.8%
	0.70-3.49	class2	8411	9.5%	991	11.8%	2584	10.6%	3009	9.5%	1827	7.5%
	3.50-17.49	class3	2979	3.4%	422	5.0%	982	4.0%	978	3.1%	597	2.5%
	17.50-49.99	class4	746	0.8%	124	1.5%	241	1.0%	226	0.7%	155	0.6%
	50.00-99.99	class5	198	0.2%	30	0.4%	58	0.2%	74	0.2%	36	0.1%
	> = 100	class6	80	0.1%	16	0.2%	21	0.1%	32	0.1%	11	0.0%
		Total	88716	100.0%	8405	100.0%	24461	100.0%	31650	100.0%	24200	100.0%
Moth												
	<0.35	class0	63857	72.0%	5826	69.3%	17490	71.5%	22859	72.2%	17682	73.1%
	0.35-0.69	class1	8079	9.1%	788	9.4%	2169	8.9%	2880	9.1%	2242	9.3%
	0.70-3.49	class2	13113	14.8%	1345	16.0%	3742	15.3%	4621	14.6%	3405	14.1%
	3.50-17.49	class3	3519	4.0%	420	5.0%	1018	4.2%	1245	3.9%	836	3.5%
	17.50-49.99	class4	152	0.2%	26	0.3%	42	0.2%	45	0.1%	39	0.2%
	50.00-99.99	class5	1	0.0%	0	0.0%	0	0.0%	1	0.0%	0	0.0%
	> = 100	class6	0	0.0%	0	0.0%	0	0.0%	0	0.0%	0	0.0%
		Total	88721	100.0%	8405	100.0%	24461	100.0%	31651	100.0%	24204	100.0%

## Discussion

This is the first report of maternal and paternal allergy-related profiles based on parental data extracted from questionnaires and blood samples taken during pregnancy, obtained from a nationwide population-based study across Japan. We report the lifetime prevalence of allergic diseases and allergen-specific IgE (>0.35 UA/mL) among parents. Our study participants reflects a real-world evaluation of allergic diseases and IgE sensitization among Japanese parents aged 20–40 years old. Around half of the study population had allergic diseases. In particular, more than 70% of the women in this cohort participated in blood sampling to detect serum IgE levels.

According to the EuroPrevall birth cohort study based on self-reported doctor’s diagnoses of allergic diseases (asthma, allergic rhinitis, and/or eczema) from nine European countries [8], the lifetime prevalence of allergic diseases among mothers was 51% in the United Kingdom; these rates for fathers were 40.1% in the United Kingdom. We found a high prevalence of allergic diseases among the Japanese population, comparable to those of the United Kingdom. The interaction between different factors, such as genetic and environmental ones, and the development of allergies in children should be effectively investigated as part of the JECS.

A nationwide cross-sectional study among 8,762 women aged 20–44 years in Japan conducted in 2006 and 2007 reported a lifetime prevalence of asthma of 11.0% [9]. The prevalence of asthma in 2006 and 2007 was nearly the same as that found in our study population. Taken together, these findings show that the prevalence of asthma has remained unchanged in Japan and seems to have reached a plateau.

It is well known that Japanese cedar pollen is a major allergen in Japan that is responsible for the development of allergic rhinitis. According to previous data, the estimated prevalence of Japanese cedar pollinosis has steadily increased over time, representing over 30% among adults aged 30–44 years [10]. Similarly, more than 30% of the parents included in our study also reported suffering from allergic rhinitis or hay fever, although the causal allergen was undetermined. This observation is supported by another report stating that more than 30% of teenagers in Tokyo suffer from allergic rhinoconjunctivitis [11]. In our study, older people tended to have higher prevalence of allergic rhinitis or hay fever. The reason might be owing to different durations of exposure to allergens. Allergic rhinitis is considered one of the most important health issues in Japan for both children and adults.

Although we did not investigate the lifetime prevalence of adult AD in our study, a local epidemiological analysis conducted in Tokyo showed that the prevalence of confirmed AD was 9.3% in women and 5.1% in men aged 20–69 years [12]. Another study reported that the prevalence of AD among adults aged 20–69 years, according to the U.K. Working Party’s diagnostic criteria [13], was 4.8% at Kinki University and 6.9% at Asahikawa University [14]. Different trends according to area, age, and time are frequently addressed in epidemiological studies [15]. Our study did not show a generational gap in the prevalence of AD, which may be stable in Japanese populations.

As for allergy to foods, younger people tended to have higher prevalence of FA compared with older adults in our study population. The trend of FA prevalence is unclear in Japan. However, according to a national survey, the prevalence of FA among elementary school-aged children has increased in Japan, from 2.8% in 1997 to 4.5% in 2013 [16]. The trend of FA in our population coincided with those results.

Interestingly, our study showed that the lifetime prevalence of contact dermatitis was different according to sex. Although we could not discriminate between irritation or allergic contact dermatitis, a previous epidemiological review reported that eczema on the hands was more common in women than in men [17]. In addition, two-thirds of patients who underwent patch testing at clinics in the United States were female [18]. The frequencies of exposure to causal products might be different between men and women. Furthermore, older generations tended to have a higher prevalence of contact dermatitis compared with younger people in our study. The reason might depend on the duration of exposure to causal products.

A previous regional epidemiological study in Fukui evaluated specific IgE antibody responses to common aeroallergens among adults aged 20–49 years in 2006 and 2007 [19]. The results demonstrated that 56% of the population had class 2 and above levels of IgE for Japanese cedar pollen, and 41% for *Der p* 1, which is similar to the findings reported in our study. A European cohort study showed that IgE sensitization in adults increased over a 10-year period, and younger generations seemed to be more sensitized and have higher IgE levels than older people [20]. It is speculated that the Japanese population may have a similar tendency to the European population. Interestingly, hen’s egg is the most common food allergen among young children in Japan [21]. However, IgE sensitization to egg white was extremely low among the pregnant women in our study. Looking into the differences by regional center, only 8.3% of mothers in the Hokkaido regional center had IgE sensitization to Japanese cedar pollen. On the other hand, 61.3% mothers in the Koshin regional center had IgE sensitization to this allergen. Japanese cedar pollen is scattered throughout the country, except in the Hokkaido region. The populations in the Hokkaido regional center were not exposed to Japanese cedar pollen and prevalence of IgE sensitization to the allergen was lower than at any other regional center. Koshin regional center is located in Yamanashi Prefecture, which is surrounded by many of the highest mountains in Japan that are covered by a large number of Japanese cedar trees. It is suggested that the populations at the Koshin regional center are highly exposed and sensitized to Japanese cedar pollen.

There are a few limitations to our study. Recall bias is a major concern in birth cohorts with self-reported questionnaires, which means that the prevalence of allergic diseases may be underestimated. In addition, while we did have a hospital-based recruitment protocol, fewer fathers agreed to participate in the JECS than mothers because most fathers usually do not visit hospitals for prenatal care. Our study was a cross-sectional study nested in a birth cohort study, thus there was a limitation to fully evaluating the differences among various age groups. Another limitation is that we could not show details of allergic diseases and specific IgE data from JECS subjects’ offspring because the JECS is ongoing and has not yet fixed or released child data. We plan to analyze the allergic features of the JECS subjects’ offspring as soon as the final data are fixed.

The prevalence of allergic diseases and IgE sensitization was different according to sex, age, and region. Sex hormones, age, and the local environment may influence the development of allergic diseases. When examining the association of chemical exposures with allergic diseases, we should take into consideration that sex, age, and area of residence are important cofounders. Because we successfully recruited about 100,000 mothers and 50,000 fathers, we intend to confirm the link between environmental exposures and childhood allergy outcome using the JECS in future studies.

## Conclusions

In summary, the JECS is a large-scale birth cohort study that has reported the allergic status of both mothers and fathers during pregnancy among a nationwide Japanese population. The aim of the JECS is to confirm the link between environmental exposures and the development of childhood allergic diseases.

## Additional files

Additional file 1: Table S1. Lifetime prevalence of allergic diseases among mothers by regional centers in the JECS (XLSX 744 kb)

Additional file 2: Table S2. Lifetime prevalence of allergic diseases among fathers by regional centers in the JECS (XLSX 19 kb)

Additional file 3: Table S3. Selected maternal serum-specific IgE titers during pregnancy by regional centers in the JECS (XLSX 26 kb)

## Abbreviations

AD: Atopic dermatitis
FA: Food allergy
JECS: The Japan environment and children’s study
